# Characterizing the rapid spread of porcine epidemic diarrhea virus (PEDV) through an animal food manufacturing facility

**DOI:** 10.1371/journal.pone.0187309

**Published:** 2017-11-02

**Authors:** Loni L. Schumacher, Anne R. Huss, Roger A. Cochrane, Charles R. Stark, Jason C. Woodworth, Jianfa Bai, Elizabeth G. Poulsen, Qi Chen, Rodger G. Main, Jianqiang Zhang, Phillip C. Gauger, Alejandro Ramirez, Rachel J. Derscheid, Drew M. Magstadt, Steve S. Dritz, Cassandra K. Jones

**Affiliations:** 1 Department of Diagnostic Medicine/Pathobiology, Kansas State University, Manhattan, Kansas, United States of America; 2 Department of Grain Science and Industry, Kansas State University, Manhattan, Kansas, United States of America; 3 Department of Animal Sciences and Industry, Kansas State University, Manhattan, Kansas, United States of America; 4 Department of Veterinary Diagnostic and Production Animal Medicine, Iowa State University, Ames, Iowa, United States of America; Sun Yat-Sen University, CHINA

## Abstract

New regulatory and consumer demands highlight the importance of animal feed as a part of our national food safety system. Porcine epidemic diarrhea virus (PEDV) is the first viral pathogen confirmed to be widely transmissible in animal food. Because the potential for viral contamination in animal food is not well characterized, the objectives of this study were to 1) observe the magnitude of virus contamination in an animal food manufacturing facility, and 2) investigate a proposed method, feed sequencing, to decrease virus decontamination on animal food-contact surfaces. A U.S. virulent PEDV isolate was used to inoculate 50 kg swine feed, which was mixed, conveyed, and discharged into bags using pilot-scale feed manufacturing equipment. Surfaces were swabbed and analyzed for the presence of PEDV RNA by quantitative real-time polymerase chain reaction (qPCR). Environmental swabs indicated complete contamination of animal food-contact surfaces (0/40 vs. 48/48, positive baseline samples/total baseline samples, positive subsequent samples/total subsequent samples, respectively; *P* < 0.05) and near complete contamination of non-animal food-contact surfaces (0/24 vs. 16/18, positive baseline samples/total baseline samples, positive subsequent samples/total subsequent samples, respectively; *P* < 0.05). Flushing animal food-contact surfaces with low-risk feed is commonly used to reduce cross-contamination in animal feed manufacturing. Thus, four subsequent 50 kg batches of virus-free swine feed were manufactured using the same system to test its impact on decontaminating animal food-contact surfaces. Even after 4 subsequent sequences, animal food-contact surfaces retained viral RNA (28/33 positive samples/total samples), with conveying system being more contaminated than the mixer. A bioassay to test infectivity of dust from animal food-contact surfaces failed to produce infectivity. This study demonstrates the potential widespread viral contamination of surfaces in an animal food manufacturing facility and the difficulty of removing contamination using conventional feed sequencing, which underscores the importance for preventing viruses from entering and contaminating such facilities.

## Introduction

Federal regulations recognize animal feed as food and an important part of our national food supply. Recent changes in legislation through the Food Safety Modernization Act, along with evolving consumer demands, are placing greater emphasis on the role of animal food in the farm-to-fork food safety system [1]. Recently, porcine epidemic diarrhea virus (PEDV), a swine pathogen present in other parts of the world, was identified for the first time in the United States [2, 3]. The introduction of PEDV into U.S. herds was remarkable because of the sheer magnitude of infectivity and impact on animal health and welfare [4, 5]. Nonetheless, it was also significant because PEDV is one of the first viral pathogens confirmed transmissible in animal food. In one proof-of-concept study, suspected particulates of animal food and dust was found infectious [6]. Potential routes of viral introduction into the animal food manufacturing process have been identified [7]. Therefore, there is potential for viral contamination of animal food manufacturing facilities [8]. However, there is no available data describing the transmission of viruses in either animal or human food manufacturing facilities, nor are there established procedures to reduce or eliminate viral contamination on food-contact surfaces. This is particularly concerning because a proof-of-concept procedure proved elimination of PEDV RNA in an animal food manufacturing facility was challenging, and extreme decontamination measures including chemical disinfectants and heat were necessary [8]. More information is needed to understand how a food-transmitted virus interacts with a manufacturing environment in order to ensure both animal and human health. Therefore, the objective of this study was to 1) characterize the extent of viral contamination in an animal food manufacturing facility and 2) test a proposed control method, feed sequencing, to decrease viral decontamination on animal food-contact surfaces as measured by quantitative real-time PCR (qPCR) and infectivity by pig bioassay.

## Materials and methods

The animal food manufacturing portion of the experiments was conducted at the Kansas State University Cargill Food Safety Research Center (FSRC; Manhattan, KS), a 3-story biosafety level 2 biocontainment laboratory and animal food manufacturing facility containing pilot scale animal food manufacturing equipment. Procedures were approved by the Kansas State University Institutional Biosafety Committee (Approval No. 929.3). All manufacturing procedures were replicated three times. Decontamination occurred before and after each replicate to establish baseline and confirmed negative by the absence of PEDV RNA on animal food-contact and non-food contact surfaces as measured by qPCR as previously described [8].

The portion of the experiment evaluating infectivity in animals was conducted at Iowa State University. Procedures were approved by the Iowa State University Institutional Animal Care and Use Committee (Approval No. 1-16-8168-S).

### Preparation of inoculum

Virus isolation, propagation, and titration were performed in Vero cells (ATCC CCL-81) as previously described [9]. The U.S. PEDV prototype strain cell culture isolate USA/IN19338/2013 cell passage 8 was used to inoculate food in this study. The stock virus titer contained 4.5 x 10^6^ TCID_50_/ml, with a corresponding qPCR cycle threshold (Ct) value of 11. The virus was divided into three 500 ml aliquots that were stored at -80°C, with one aliquot used per replication. For each replication, an aliquot was thawed overnight at 4°C, added to 4.5 kg of animal food using mixing procedures previously established [10] to form the animal food inoculum.

### Animal food manufacturing

A corn-soybean meal-based diet with a composition typically fed to adult swine was manufactured at the Kansas State University O.H. Kruse Food Technology Innovation Center (Manhattan, KS) (Table 1). A subsample of the animal food was obtained prior to inoculation for each replication and confirmed PEDV negative by qPCR. Prior to inoculation, 50 kg of the animal food was mixed in a 0.113 m^3^ electric paddle mixer (H. C. Davis Sons Manufacturing model# SS-L1; Bonner Springs, KS) that was previously validated to mix a 50 kg batch of animal food with CV less than 10%, as per standard mixing efficiency protocol [11]. The animal food was mixed for 5 min, then discharged at a rate of approximately 4.5 kg/min into the conveyor (Universal Industries, Cedar Falls, IA) that carried 74 buckets (each 114 cm^3^) of animal food. The animal food was conveyed and exited through a downspout into biohazard bags.

**Table 1:** Diet composition of porcine epidemic diarrhea virus (PEDV) inoculated animal food, as fed basis.

Ingredient, %	Composition
Corn	79.30
Soybean meal, 46.5% CP	15.70
Choice white grease	1.00
Calcium phosphate (monocalcium)	1.40
Limestone	1.15
Salt	0.50
_L_-Threonine	0.03
Trace mineral premix^a^	0.15
Sow add pack^b^	0.50
Vitamin premix^c^	0.25
Phytase^d^	0.02
Total	100.00
Formulated analysis^e^, %	
DM	91.4
CP	17.1
Crude fiber	3.7
Ca	0.78
P	0.52
Fat	3.5

^a^Each kilogram of premix contains 73 g Fe, 73 g Zn, 22 g Mn, 11g Cu, 0.198 mg I, and 0.198 mg Se.

^b^Each kilogram of premix contains 4,409 IU vitamin E, 44 mg biotin, 992 mg pyridoxine, 331 mg folic acid, 110,229 mg choline, 40 mg chromium, 9,920 mg _L_-carnitine.

^c^Each kilogram of premix contains 4,409,171 IU vitamin A, 551,146 IU vitamin D_3_, 17,637 IU vitamin E, 1,764 mg menadione, 3,300 mg riboflavin, 11,023 mg d-pantothenic acid, 19,841 mg niacin, 15 mg vitamin B_12_.

^d^High Phos 2700 GT, DSM Nutritional Products, Parsippany, NJ.

^e^One sample was analyzed by Ward Laboratories Inc., Kearney, NE.

### Inoculation of diet and animal food manufacturing

The previously-prepared 5 kg of inoculum was added to 45 kg of virus-free animal food in a 0.113 m^3^ electric paddle mixer (H. C. Davis Sons Manufacturing; Model SS-L1; Bonner Springs, KS) to form the positive control, and was mixed and discharged as described above. Four sequenced 50 kg batches (Sequence 1 to 4) of virus-free animal food were mixed and discharged after the positive control without any cleaning or decontamination between batches to mimic commercial animal food production conditions.

### Environmental observation

Prior to and after each batch of feed being manufactured, environmental surfaces were swabbed using large foam-tipped disposable swabs (World Bio-Products LLC, Woodinville, WA) that were pre-wetted with 2 ml of phosphate buffered saline. To collect samples, a clean pair of disposable gloves was worn, each swab opened aseptically, and rubbed across the desired surface. Swabs were then capped and placed in a cooler with ice until analyzed.

**Fig 1:**
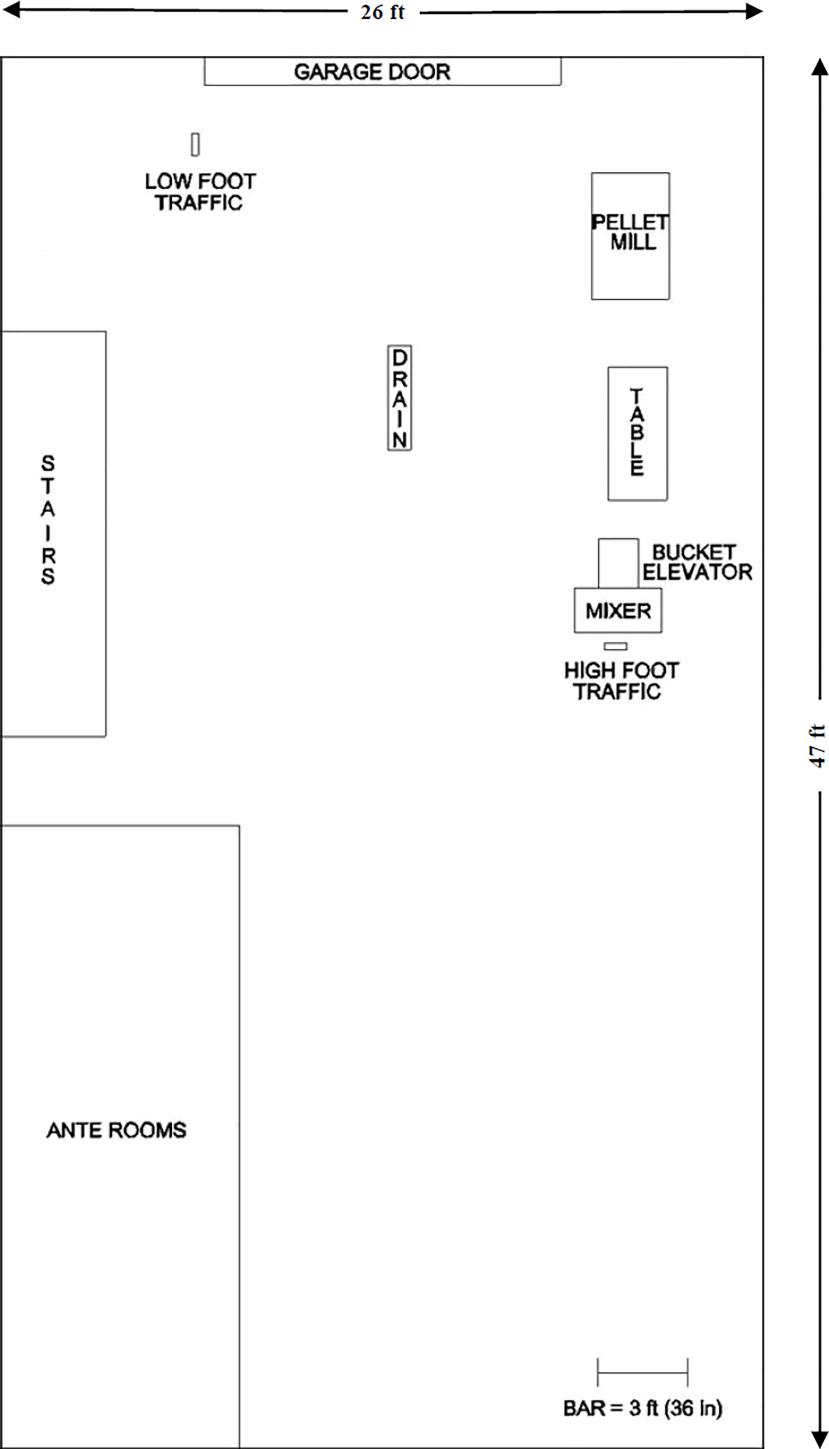
Layout of research facility. Designated areas swabbed for PEDV qPCR analysis include high and low foot traffic areas (concrete), drain (concrete), garage door (metal), pellet mill (equipment), table ledge (metal), conveyer (equipment), and food mixer (equipment). Not shown are rubber boot bottoms (rubber).

Designated locations were sampled as illustrated in Fig 1. At each location, surfaces were outlined in heat-stable marker to form 5 equal-sized subsample areas. One randomly selected area was swabbed at each location before manufacturing (baseline), and after each manufactured batch of animal food. Designated surfaces included the drain, floor with high foot traffic, floor with low foot traffic, garage door, table ledge, mixer paddle, mixer interior lid and mixer interior of bottom, boots worn during the experiment, the interior of 4 plastic conveyer buckets (one swab each) and 4 rubber belt areas (one swab each) adjacent to the chosen buckets. Swabs were categorized by surface (metal, concrete, plastic vs. rubber) within zone (animal food-contact vs. non-animal food contact). Immediately after completion of the study, supernatant from swabs were transferred to 96-well plates and plates were stored frozen at -80°C until initiation of the bioassay. The plates were then thawed at room temperature, supernatant was pooled according to replicate and treatment for each pig and were then stored at 4°C overnight until used for bioassay the next day (0 DPI).

### Pig study

Eighteen pigs were purchased from a conventional breeding farm and delivered to the Iowa State University Laboratory Animal Resource (LAR) facilities. All pigs were administered an intramuscular dose of ceftiofur (Exede; Zoetis, Florham Park, NJ) per label instructions upon arrival and confirmed negative for PEDV, porcine delta coronavirus (PDCoV), transmissible gastroenteritis virus (TGEV) and porcine rotaviruses (groups A, B, and C) by virus specific qPCR on rectal swabs. In addition, pigs were confirmed PEDV antibody negative by fluorescent foci neutralization serologic analysis performed at South Dakota State University Veterinary Diagnostic Laboratory (SDSU VDL).

A bioassay was conducted 11 months after animal food preparation and sample collection. A total of 6 rooms (3 pigs per room) were assigned to swabbed dust samples collected from the conveyer after production of each animal food treatment (1 negative control room and 5 challenge rooms). Pigs were blocked by weight, then randomly divided into groups of 3 per room. Rooms had independent ventilation systems and solid flooring that was minimally rinsed to reduce PEDV aerosols. Pig were fed liquid milk replacer (Esbilac; PetAg, Hampshire, IL) and commercially pelleted diet (All Natural Starter 2; Heartland Co-op, Alleman, IA). Pigs had ad libitum access to food and water at all times.

After 2 days of acclimation, each pig was administered the dust suspension from swabbed surfaces by orogastric gavage using an 8−gauge French catheter and 60 ml syringe (8 ml/pig), which marked day 0 post inoculation (0 DPI). The 8 ml aliquot combined eight 1-ml dust suspensions sampled from 4 buckets and 4 adjacent belt areas after manufacturing each food treatment from one replicate. Thus, each pig represented 1 of 3 replicates per treatment and each room represented each treatment.

Rectal swabs were analyzed from all pigs on -2, 0, 2, 4, 6, and 7 DPI. Swabs were submerged into 1 ml phosphate buffered saline (PBS, 1 × pH 7.4) immediately after collection and submitted to Iowa State University Veterinary Diagnostic Laboratory (ISU VDL) for PEDV RNA by qPCR. All pigs were euthanized at 7 DPI for necropsy by intravenous overdose of pentobarbital sodium solution as per label instructions (Fatal-Plus; Vortech Pharmaceuticals Ltd, Dearborn, MI). At necropsy, an aliquot of fresh cecal contents was submitted for PEDV qPCR to ISU VDL.

### RNA extraction and quantitative PEDV RT-PCR (qPCR)

Dust samples from swabs were tested at Kansas State University Molecular Diagnostics Development Laboratory (Manhattan, KS) for PEDV using a PEDV spike (S) gene-based qPCR. Nucleic acids were extracted from a 50 μL sample of supernatant. Automated extraction was carried out on a KingFisher magnetic particle processor (Thermo Scientific, Waltham, MA) using a MagMAX-96 Viral RNA Isolation Kit (Life Technologies, Grand Island, NY). All manufacturer’s instructions were followed, with the exception of a final elution volume of 60 μl. Each 96-well extraction run included an extraction positive control (PEDV stock virus) and an extraction negative control (1x PBS). Four μl of RNA template was used in qPCR setup in a 20 μl reaction using a real time RT-PCR kit (Path-ID Multiplex One-Step RT-PCR Kit; Thermo Scientific, Waltham, MA). Amplification reactions were conducted on a CFX96 Touch Real-Time PCR Detection System (Bio-Rad Laboratories, Hercules, CA). The thermal cycling parameters were: 10 min reverse transcription at 48°C, 10 min of reverse transcriptase inactivation/initial denaturation at 95°C followed by 45 cycles of 10 sec at 95°C and 40 sec at 60°C.

Animal samples and samples for bioassay were tested for PEDV using a previously described PEDV nucleocapsid (N) gene-based qPCR [12]. Nucleic acids were extracted from the stock virus (50 μl), bioassay inoculum (100 μl), and rectal swabs (100 μl), and eluted into 90 μl of elution buffer using an RNA/DNA kit (MagMAX Pathogen RNA/DNA Kit; Thermo Scientific, Waltham, MA) and a Kingfisher-96 magnetic particle processor (Thermo Scientific, Waltham, MA) following the manufacturer’s instructions. Five μl of RNA template was used in qPCR setup in a 25 μl reaction using a real time RT-PCR kit (Path-ID Multiplex One-Step RT-PCR Kit; Thermo Scientific, Waltham, MA). Amplification reactions were conducted on an ABI 7500 Fast instrument (Thermo Fisher Scientific, Waltham, MA) following previously described procedures [12].

### Statistical analysis

Swabs were categorized as animal food-contact and non-animal food-contact surfaces. Within animal food-contact surface, Ct analysis of the metal mixer, plastic conveyer buckets, and rubber conveyer belt were performed using PROC GLIMMIX (SAS Institute, Inc., Cary, NC). Within animal food-contact surface, the statistical model evaluated the effect of treatment (negative, positive, sequence 1, sequence 2, sequence 3 and sequence 4) and surface (metal mixer, plastic conveyer buckets, and rubber conveyer belt) and the associated interaction. Each swab was classified from treatment and surface type. The LSMEANS procedure compared surface type among treatments within animal food-contact surfaces by pairwise comparison. The non-animal food-contact surfaces were reported in the results text using descriptive statistics; non-animal food-contact swabs were organized by surface type (metal garage, metal tabletop, concrete floor, and rubber boot bottoms worn during the experiment) among treatments. Samples considered negative by qPCR were evaluated as a value of 45 in the statistical model. Results were considered significant at *P* ≤ 0.05.

## Results

As expected, all animal food-contact negative control swabs were qPCR negative (Table 2). After the positive treatment was manufactured, the count of qPCR positive swabs increased to 100%. After sequence 1, 100% of swabs remained qPCR positive, and the mean Ct of samples from the metal mixer were higher (*P* < 0.05) than plastic conveyer buckets or rubber belt. After sequence 2, 67% of metal mixer swabs were qPCR positive, whereas 100% of plastic conveyer buckets and rubber belt swabs were qPCR positive. After sequence 3 and four, 44% of metal mixer swabs were qPCR positive and 100% of plastic conveyer buckets and rubber belt were again qPCR positive. For mean Ct values, there was an animal-food contact surface × treatment interaction (*P* < 0.05). After manufacturing of the positive batch of animal food, the mean Ct value of the metal mixer increased through sequence 3, however there was no significant Ct or further improvement after sequence 4. Unlike the metal mixer, the mean Ct value of surfaces from the conveyor rubber belt did not change after sequencing animal food after manufacturing of the positive animal food treatment. For the plastic conveyer buckets, after sequence 1, there was a Ct increase (*P* < 0.05) followed by another increase after sequence 2, however sequence 2 and 3 did not differ. Additionally, after sequence 4, Ct values did not differ after sequence 3, however was lower (*P* < 0.05) than Ct values after sequence 2.

**Table 2:** Effect of contamination on animal food-contact zone and their types after porcine epidemic diarrhea virus (PEDV) inoculated animal food manufacturing†.

	Treatment					
Item	Negative	Positive	After sequence 1	After sequence 2	After sequence 3	After sequence 4
Contact Zone, Detectable RNA/Total^‡^						
Animal food-contact						
Metal mixer^¶^	0/9	9/9	9/9	6/9	4/9	4/9
Plastic conveyor bucket^#^	0/12	12/12	12/12	12/12	12/12	12/12
Rubber conveyor belt^††^	0/12	12/12	12/12	12/12	12/12	12/12
Swab, Ct*						
Metal mixer	45.0^a^	29.2^h^	33.9^d^^e^	38.2^c^	40.7^b^	40.5^b^
Plastic conveyor buckets	45.0^a^	30.8^h^	32.1^e^^f^^g^	34.2^d^	32.8^d^^e^^f^	32.1^e^^f^^g^
Rubber conveyor belt	45.0^a^	30.8^g^^h^	31.5^f^^g^	31.5^f^^g^	32.2^e^^f^^g^	32.1^e^^f^^g^

^abcdefg^Superscripts within a row that do not share a letter differ *P* < 0.05.

^**†**^Tissue culture fluid containing 4.5 × 10^6^ TCID_50_/ ml of PEDV was inoculated into 45 kg of PEDV negative food to form the positive treatment. For each negative, positive and sequenced batch, food was mixed for 5 min, discharged for 10 min into a conveyer and collected upon exit. Dust was then collected from surfaces using swabs pre-wetted with 2 ml of PBS. Equipment was not cleaned between treatments. Sequences were formed by sequentially adding 50 kg of PEDV negative food to the mixer after the prior batch was processed. This experiment was replicated 3 times. For swab Ct analysis, surface × treatment *P* <0.0001 and pooled SEM ꞊ 0.67.

^‡^Count of swabs with detectible PEDV RNA/number of swabs analyzed.

^¶^Metal includes one sample each from the mixer paddle, mixer interior lid, and mixer interior bottom.

^#^Plastic includes one swab each from 4 randomly chosen interior conveyor buckets.

^**††**^Rubber includes one sample each from 4 belt areas adjacent to chosen conveyor buckets.

^*^Mean cycle threshold (Ct) value of samples. A value of 45.0 was used for samples with no detectible PEDV RNA.

All non-animal food-contact surface baseline swabs were qPCR negative. Non-animal food-contact swabs were analyzed by surface type (metal garage, metal tabletop, concrete floor, and rubber boot bottoms worn during the experiment). Unexpectedly, in 1 of 3 repetitions, 1.7% of non-animal food-contact surface swabs were qPCR positive after the negative treatment was manufactured, although the animal food was qPCR negative. For all repetitions, after the positive treatment and after sequence 1, 89% of non-food-contact surface swabs were qPCR positive. After sequence 2, 94% of non-food-contact surface swabs were qPCR positive. After sequence 3, 89% of non-food-contact surface swabs were positive that again increased to 94% after sequence 4. The percentage of positive swabs from non-animal food-contact metal surfaces (metal garage and tabletop) varied, whereas non-animal food-contact concrete floor and rubber boot bottoms remained the same (67%, 67%, 83%, 67%, 83%; after positive, after sequence 1, after sequence 2, after sequence 3 and after sequence 4, respectively vs. 100% after positive and sequence 1 to 4, respectively). Dust suspensions from animal food-contact surfaces were challenged in pigs and failed to produce infectivity.

## Discussion

The recent enacting of the Food Safety Modernization Act (FSMA) requires animal food manufacturers to identify and control animal food safety hazards because feed is considered animal food and a part of the human food safety system [1]. Hazard characterization includes biological hazards, such as *Salmonella* spp. and *Listeria monocytogenes* [13]; however viral pathogens were not traditionally considered common biological hazards in animal food until after the introduction of PEDV to North America. Recent research identified swine food as one of many potential vectors for virus transmission, and confirmed PEDV contaminated foodstuffs may cause disease [14, 15]. While animal food is not likely the predominant vector, it was one of the remaining potential vectors for PEDV transmission that was not previously controlled by on-farm biosecurity measures. This is concerning because little is known about virus contamination during the manufacturing of animal food. Likewise, viral transmission in animal food manufacturing facilities is not well characterized, nor are tested control methods available to reduce contamination on animal food-contact surfaces. While there are no currently identified similar cases of viral transmission through the human food chain, its potential exists and information gleaned from studying PEDV transmission may be applicable if a virus impacting human health were to enter the human food manufacturing system.

For these reasons, an established protocol for monitoring viral transmission is needed to model animal and human food hazards if additional pathogenic viruses are discovered in our food supply. This is the first study of its kind to fully observe environmental contamination of an animal food-manufacturing facility during a proposed control method after manufacturing viral-inoculated swine food. Objectives were met by monitoring the extent of virus contamination in an animal food manufacturing facility and investigating a control method to decrease virus contamination on animal food-contact surfaces.

In general, environmental contamination of a virus in any food manufacturing facility has not been well-documented. In human food, norovirus is a known cause of foodborne illness with contamination presumed at point-of-service [16, 17]. However, there is little information regarding norovirus-contaminated food at the manufacturing level due to inadequate surveillance or facility control measures [18]. Even less is known about viral contamination in animal food manufacturing facilities.

The results from this study clearly demonstrate the extent of the widespread viral contamination that occurs in an animal food manufacturing facility after production of virus-inoculated animal food. All of the animal food-contact surfaces and most of the non-animal food-contact surfaces were qPCR positive when swabbed after the contaminated animal food was manufactured and remained qPCR positive after multiple batches of animal food were mixed and conveyed. Therefore, it seems that the proposed mitigation technique (feed batch sequencing) did not mitigate environmental PEDV contamination. Additionally, detectible PEDV seemed to persist on some animal food-contact surfaces, such as plastic and rubber conveyors, more than others such as metal. Previous studies have investigated the survivability of virus on inanimate surfaces and determined viral persistence in the environment can be affected by several factors including surface type [19–21]. Additionally, different surface types can have different characteristics such electrostatic, hydrophobic or ionic strength which may impact virus detectability on these surfaces [22, 23]. For example, it has been reported that electrostatic forces impact virus attachment to lettuce [24]. Therefore, it is possible that physical properties contributed to the persistence of PEDV on animal food-contact surfaces sampled in the current study. This is interesting because most animal food manufacturing equipment have been designed for electrical efficiency and physical cleanout, but not sanitization. For example, plastic conveyer buckets are preferred not only because they are light and more energy efficient, but they are also safer for workers due to elimination of sparking that is a concern with sheeted metal buckets [25].

In pet food manufacturing, equipment surfaces are easy-to-clean with non-porous equipment surfaces selected in order to prevent biofilms or the prevalence of *Salmonella* spp. or *Listeria monocytogenes*. They are also routinely sanitized with steam or chemical sanitizers. Other animal food manufacturing facilities have not selected equipment for these purposes due to previously limited risk for biological hazards. Thus, other strategies, such as use of chemical additives in animal food, may need to be employed to reduce cross-contamination of PEDV in animal food or ingredients [26].

Alternatively, the difference in rate of contamination between the metal mixer or plastic and rubber in the conveyor may be due to equipment design. For example, mixers are typically designed to self-clean with little residual material from one batch to the next compared to conveyors. This is particularly true of bucket elevators, which is the conveyor type used in this experiment. The large rubber belt of a bucket elevator is suspended vertically, and plastic buckets convey feed upward until the feed is flipped from the buckets into a discharge chute. The boot pit, which is the area at the bottom of the bucket elevator, must be large enough for buckets to clear the bottom without coming into contact with the guard or cover. This area typically fills with residual feed and may lead to batch-to-batch cross contamination, which has been demonstrated by carryover of animal drugs [27]. Therefore, it is reasonable to extrapolate that batch-to-batch carryover of feed residue may also exist when the hazard is an undesirable microorganism.

This research concludes that differences exist in viral contamination rates on different equipment surfaces, which may be due to differences in surface type, equipment design, or other phenomena. Regardless of the source of these differences, animal food manufacturing facilities at risk for PEDV contamination should consider these findings when choosing manufacturing equipment. The results of the current experiment are applicable to other species of animal food and to human food manufacturing facilities because entry of a viral pathogen may cause widespread contamination that is difficult to eliminate. Even with wet chemical cleaning and facility heating, PEDV proved difficult to decontaminate from our facility [8]. This is concerning because extreme methods were used, which are impractical in commercial animal food manufacturing settings.

In the current study, environmental surfaces were swabbed for dust after production of PEDV inoculated animal food and animal-food contact surfaces were evaluated for infectivity. A previous proof-of-concept-study demonstrated that animal food dust can be infectious [6]. Although the exact cause for lack of infectivity in this study is unknown, storage time may have impacted virulence in these samples since long-term low temperature storage has been reported to affect virus fitness and recoverability [28–30]. Additionally, although the minimum infectious dose is low in animal food [10], perhaps not enough viral particles were collected by or eluded from swabs to cause an infection in the present study. Although we were unsuccessful at finding evidence of infectivity in this study, the hypothesis that environment dust is infectious after animal food batch sequencing is still conceivable and remains to be proven.

Another result from this study is that some non-food contact swabs from a repetition were qPCR positive after the negative animal food was manufactured, although importantly, animal food tested was qPCR negative. We hypothesize this genetic material remained on the boot due to inadequate cleaning after a previous replicate and was tracked then detected on the concrete floor. Due to the chemical cleaning between repetitions, the viral material should not have been infective [31]. However, we believe contaminated rubber boot bottoms worn during the experiment helped track and spread the virus as genetic material was consistently detected on concrete floor surfaces. This underscores the importance of foot traffic biosecurity in any facility, including animal food manufacturing facilities [7]. This is especially true as demonstrated in one study, PEDV and porcine deltacorona virus was detected from multiple locations within and around animal food manufacturing facilities [32] which again illustrates foot traffic can be a biosecurity problem. Therefore, key implications from these findings is that foot traffic should be limited across receiving pits or in hand-add areas that have direct access to animal food contact equipment and boots should be cleaned regularly to minimize risk of inadvertent contamination.

As the current study demonstrates, widespread contamination of PEDV occurred and was detected on most surfaces. Material collected from dust collection systems and sweepings should be collected and disposed instead of added to the product flow as per traditional measures [7, 33]. Therefore, animal food manufacturing facilities should re-consider before using dust collected from dust disposal systems and instead consider including procedures to minimize and control dust since it could be a vector of possibly infectious PEDV. Again, once an animal food manufacturing facility is contaminated with an undesired microorganism, it is difficult to eliminate and thus prevention protocols should be implemented [34, 35].

In conclusion, this study clearly demonstrates widespread contamination occurred in an animal food manufacturing facility after PEDV swine food production. Furthermore, the proposed mitigation method of feed batch sequencing was not effective to reduce environmental contamination, although the potential impact of PEDV contamination and importance to prevent virus entry in such facilities was better understood. It is concerning once an animal food manufacturing facility is contaminated with PEDV, it appears to harbor PEDV until chemically cleaned. This research indicates animal food manufacturing facilities potentially contaminated with PEDV can be a central point for virus transmission and the quantification for this risk should be assessed. As a result, the practicality of decontamination is a new challenge facing our animal food manufacturing facilities.
